# Cytogenetic changes in *Rosa spinosissima* L. and *Leymus angustus* (Trin:) Pilg. growing under radioactive contamination conditions at the Semipalatinsk nuclear test site

**DOI:** 10.1371/journal.pone.0324860

**Published:** 2025-05-22

**Authors:** Kyrmyzy Minkenova, Arailym Serik, Andrey Panitskiy

**Affiliations:** Institute of Radiation Safety and Ecology, NNC RK, Kurchatov, Kazakhstan; Universiti Teknologi Malaysia - Main Campus Skudai: Universiti Teknologi Malaysia, MALAYSIA

## Abstract

In this study, we investigated the cytogenetic effects of exposure to chronic radioactive contamination on the populations of *Rosa spinosissima* L. and *Leymus angustus* (Trin.) Pilg. growing in fields affected by radioactive water streams from the ‘Degelen’ test location of the Semipalatinsk test site. The results revealed that the radiation dose absorbed by these plants varied from 108 to 1,150 µGy/day, depending on the sampling points of the plants. The main exposure dose received by the plants was from ^90^Sr and ^137^Cs. In both plant species, chromosomal aberrations were the main contributors to the range of cytogenetic effects (double bridges and double fragments). The proportions of chromosomal aberrations were the highest among all cytogenetic effects at 42 and 54% in *R. spinosissima* and *L. angustus*, respectively. A linear relationship was established between the increase in the frequency of aberrant cells and the increase in the rate of radiation dose absorption in *R. spinosissima* for the entire range of the absorbed doses in question up to 1,129 µGy/day and in *L. angustus* for the range of absorbed doses from 152–583 µGy/day.

## Introduction

From 1949 to 1989, 456 nuclear tests were conducted at the Semipalatinsk test tite (STS), during which more than 600 nuclear charges were detonated. Of the total conducted tests, 30, 88, and 348 were ground, air and underground tests, respectively. The various types of nuclear tests conducted in this area exhibited specific features and exerted different effects on the environment and public health [1–3]. Therefore, the STS area can be considered a ‘field laboratory’, which differs from other test sites by its arid climate and wide range of radionuclides, as well as by the presence of local plots with a dominant contribution from different types of radiation to the generation of a radiation burden on organisms. Field-based studies differ from laboratory work in the complexity and interpretation of results. Radioactive contamination can lead to the death of individual species and entire populations, thus disrupting the ecological balance. The uniqueness of STS makes it possible to perform radiobiological studies of biogeocenoses exposed to chronic radiation against the critical research objects, which, being the basis of the food chain, can experience the effects of various stress factors earlier than the organisms at higher trophic levels [4]. Earlier, we conducted studies of the cytogenetic effects of exposure to chronic radiation on wild plants growing at the ‘4A’ site, where the tests of radiological warfare agents had been conducted [5–7]. The site ‘4A’ is notable for high levels of ^90^Sr contamination. Therefore, it would be equally important to study the cytogenetic effects of exposure to chronic radioactive contamination on wild plants growing in areas affected by radioactive water streams from the test adits at the ‘Degelen’ test location.

**Fig 1:**
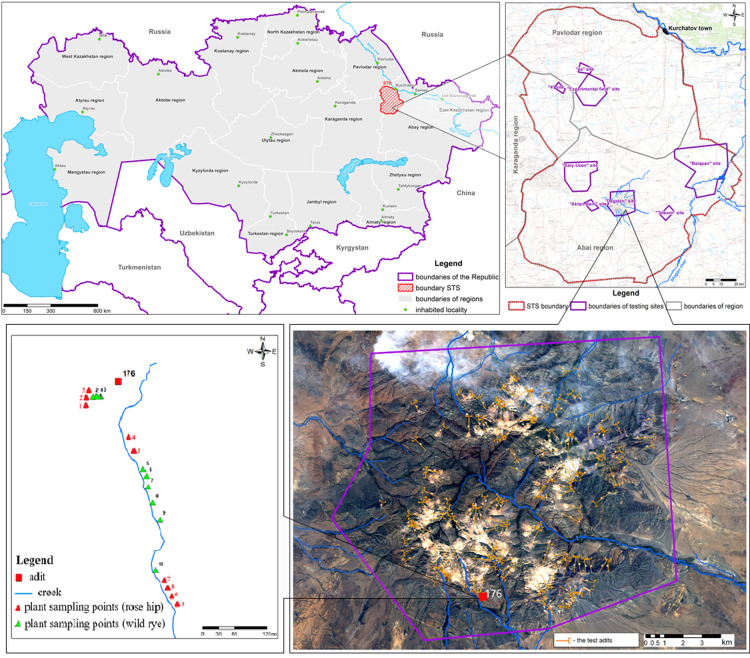
Layout of the sampling points for the soil, plant, and seed samples collected from the ecosystem of exposed to the water stream from tunnel 176 at the Degelen test location [10].

The ‘Degelen’ test location is at a mountain range with the same name south of the STS (Fig 1). It has a total area of approximately 350 km^2^. The Partial Nuclear Test Ban Treaty, signed in 1963, initiated the creation of this site to conduct underground tests in horizontal mining workings and adits. Nuclear weapons blasts conducted in the adits of the ‘Degelen’ mountain range from 1961 to 1989 led to radioactive contamination not only inside the adits, but also on the surface [8,9].

In this study, we aimed to analyse cytogenetic effects of chronic radioactive exposure on the populations of *Leymus angustus* (Trin.) Pilg. and *Rosa spinosissima* L. growing in an ecosystem affected by a radioactive contaminated water stream from test adit 176 at the ‘Degelen’ test location ofSTS.

## Materials and methods

### Field activities

The field-work at STS was conducted under the available licence for activities in the areas of former nuclear test sites contaminated by nuclear blasts. The investigation was conducted during the seed-ripening period of the plant species of interest, i.e., in late August and July for *Rosa spinosissima* L., and, *Leymus angustus* (Trin.) Pilg*.,* during summer. These plant species grow in the valleys of all creeks at the Degelen test location [11], and therefore, were chosen for the study. In addition, these plants differ in their life forms. *Rosa spinosissima* L. is a brushwood, species, whereas *L. angustus* (wildrye) is a herbaceous plant.

To investigate the cytogenetic indicators of plants growing in radionuclide-contaminated STS areas, at radioactive water stream ecosystem was selected at adit 176 in the valley of Baitles Creek. A schematic of the location of adit 176 is shown in Fig 1. The choice of adit 176 was based on the available data that the water stream released from adit 176 carries large amounts of ^137^Cs and ^90^Sr [8]. The location of adit 176 also ruled out the influence of other adits on the radiological situation. To determine the effects of different concentrations of radionuclides and radioactive elements on the cytogenetic indicators, research sites (sampling points) with a high probability of the elevated contents of radioactive elements and radionuclides were chosen. Therefore, during the field work, the equivalent dose rate (EDR) and the β –particle fluence were measured with a dosemeter-radiometer MKS-АТ6130 (OJSC MNIPI, Belarus) at different field locations. Radiometric parameters were measured 0.3 cm above the soil surface.

### Sampling locations and techniques

Nine research points were located on *R. spinosissima* and 10 on *L. angustus* in different sections of the water stream from Tunnel 176. The layout of the sampling points is presented in Fig 1. The points are selected based on the occurrence of the species of interest, as well as based on the data on the distribution of the β- particles fluence and EDR. Seeds from each site were selected for cytogenetic analysis. The remaining aboveground parts of the plants were mown from a 1 m^2^ area for radionuclide and elemental analyses. The aboveground parts of *R. spinosissima* were sampled based on the sufficiency calculation for the analysis (no more than 300 g), and duplicate soil and plant samplings were performed to determine their radionuclides contents.

### Laboratory research

#### Radionuclide analyses.

Soil samples were sieved through a 1 mm mesh and dried to a constant weight. The activity concentrations of ^137^Cs and ^241^Am in the dried soil samples were determined with a γ-spectrometer Canberra GX-2020 (CANBERRA, USA). The minimum detectable activities (MDA) of ^137^Cs and ^241^Am were 2.0 × 10^–1^ and 2.4 × 10^–1^ Bq kg^–1^. For γ-spectrometer calibration, the calibrating sources such as IAEA-RGK-1 potassium sulphate and, IaEa-RGTh-1 thorium ore, IAEA-RGU-1 diluted uranium ore were used. Measurements were performed according to a previously described procedure [12].

The activity concentration of ^90^Sr was determined with a β-spectrometer ‘Progres-BG’ (DOZA, Russia) [13]. To that end, following the γ-spectrometric analysis, a 15 g subsample was collected from each soil sample by quartering. The activity concentrations of ^90^Sr were measured directly with the β- spectrometer ‘Progres-BG’ on an aluminum mould. The exposure time was at least 20 min (MDA: – 100 Bq kg^–1^). The accuracy of the activity concentrations of ^90^Sr was verified by the periodic measurements of the calibration reference source of ^22^Na.

To determine the activity concentrations of ^239+240^Pu in the soil samples, they were first subjected to radiochemical decomposition to obtain counting samples [13]. Subsequently, the activity concentrations were measured using the α-spectrometric technique with the α-spectrometer Alpha-Analyst (CANBERRA, USA) fitted with a solid-state passivated implanted silicon detector, and the MDA of ^239+240^Pu was 1.2 × 10^–1^ Bq kg^–1^. The α-spectrometer was calibrated using a calibration reference source of ^239^Pu manufactured by Source Inc. (Santa Fe, USA) provided with a calibration certificate Ref. # 100060.

The activity concentrations of radionuclides in the plants were determined in prewashed dry and crushed plant samples according to standard guidelines [13,14] using certified laboratory instrumentation. The measurement errors for the activity concentrations of ^137^Cs and ^241^Am mostly did not exceed 10–20%, whereas those for the activity concentrations of ^90^Sr and ^239 + 240^Pu did not exceed 15–25% and 30%, respectively.

#### Mapping.

Map documents were prepared using the software package ArcGIS. These documents were based on the digitised maps of the Republic of Kazakhstan acquired from the Republican Public State Enterprise ‘National Mapping and Geodesic Fund’ of the Committee for Geodesy and Map-Making of the Ministry of Digital Development, Innovations and Aerospace Industry of the Republic of Kazakhstan (State Procurement Contract No. 02–19/122 dated 04/28/2020).

#### Determination of the concentrations of chemical elements.

To determine the concentrations of chemical elements, plant samples were subjected to autoclave digestion following a standard procedure [15]. Soil particles were removed from plant samples (with distilled water). Thereafter, they were air-dried and sequentially crushed to 5–8 mm lengths (using stainless-steel scissors). Using a quartering technique, a medium 100 g subsample was collected from the resulting sample, which was additionally milled on an electric Grindomix GM 200 (stainless-steel blade), followed by collecting a final 5 g subsample for analysis. The subsample was placed in the fluoroplastic insert of the autoclave, wetted in a 1 ml of water, followed by the addition of 6 ml of concentrated HNO_3_ and 1 ml of 30% Н_2_О_2_. The resulting solution was left to stand for 40 min and then decomposed for 4 h in the autoclave placed in an oven heated to 160 °С. Following the autoclave digestion step, the sample was cooled down and quantitatively transferred to a volumetric test tube making up the to 15 ml with 1% HNO_3_ solution. The resulting solution was diluted in a 1:10 ratio and analysed for the elements of interest using inductively coupled plasma mass spectrometry with a quadruple mass spectrometer Perkin Elmer SCIEX Elan 9000 (Perkin-Elmer, USA) [15]. Using this technique, the concentrations of elements, such as V, Cr, Mn, Co, Ni, Cu, Zn, As, Sr, Cd, Cs, Pb and U having the detection limits of 0.01–100 µg ^–^l and the uncertainties of 10–20% were determined. For the calibration of the spectrometer, calibration solutions with 10 and 20 µg l − ^1^ concentrations of the analytes were used. Calibration graphs were plotted using the multi-element solutions of reference standards containing metals (Perkin Elmer, USA), with a certified value of 10 mg l^–1^ of metal content and an uncertainty of 0.5% in this certified value (dilution factor k = 2).

### Cytogenetic analyses of plant samples

For the cytogenetic analyses, we followed the procedure for the cytogenetic analyses of chromosomes during the first mitosis of the meristematic rootlets of germinating plant seeds [16]. Before the cytogenetic analyses, the office studies of the collected plant seeds, including their clean-up and stratification, were conducted [16]. The seeds were cleaned of all damaged seeds, stem fragments and husks, dried at room temperature (18–22°C) and then subjected to cold stratification, for which, they were stored for 30 days at a temperature of 1–5°C in a refrigerator. Subsequently, following the preparatory work, these air-dried plant seeds were placed on a wet filter paper in Petri dishes and sprouted in a thermostat MIR-253 (Sanyo, Japan) at 18–25°C.

The samples for the cytogenetic analyses were observed under an ‘Axio Imager M2 microscope (Zeiss, Germany) fitted with the objectives of 100× (oil immersion), 40× and 10 × lens magnifications.

During cytogenetic analyses, the frequency of chromosomal aberrations in the apical meristems of germinating seed rootlets was investigated. The chromosomes were analysed for abnormalities, such as the formation of bridges and fragments and chromosome lagging and leading.

The percentage of aberrant cells or the frequency of chromosomal aberrations (*F,* %) was determined using Equation (1) as follows:


$$F(\;\%)=\frac{A\times 100}{N},$$ (1)

where A is the number of aberrant cells, and

N is the total number of cells viewed.

The range of aberrations (*S,* %) was determined using Equation (2) as follow:


$$S(\;\%)=\frac{D\times 100}{N},$$ (2)

where D is the number of aberrations of a certain type, and

N is the total number of cells viewed.

The fraction of aberrations for each type (*В,* %) of the total number of all aberrations was derived using Equation (3) as follows:


$$B(\;\%)=\frac{D\times 100}{C},$$ (3)

where *C* is the total number aberrations.

Data on chromosome aberrations obtained from the cytogenetic studies were statistically processed [17]. When analysing the frequency and range of chromosomal aberrations in root meristem cells, both the normal and aberrant cells, i.e., cells with normal ana-telophase stages and those with aberrations these stages, respectively, were taken into account. Differences in the frequency of cytogenetic disorders depending on the absorbed radiation dose were analysed using statistical analysis of qualitative traits [18].

In total, 113 and 460 specimens of apical meristems from the seed rootlets of *R. spinosissima* and *L. angustus*, respectively, were examined and the number of ana-telophase cells analysed for the two species was over 1,500 and 10,738, respectively.

### Radiation burden calculations

The rate of radiation dose exposure for a plant is estimated by summing up the rates of internal and external radiation dose exposure. The first and second constituents are attributed to radionuclides contained in plants and in the soil, respectively [19].

The rate of radiation dose exposure for a plant is computed using Equation (4) as follows:


D=∑(A×d), (4)

where А (Bq kg^−1^) is the activity concentration of a radionuclide in plants or soil in case the rates of internal or external radiation dose exposure, respectively, are calculated, and

*d* is the radiation dose factor (µGy/day)/(Bq kg^-1^) of the internal or external exposure of a plant [18].

## Results and discussion

### Radiometric indicators

The measurements of radiometric parameters at the sampling points for the soil, plant and seed samples are listed in Table 1.

**Table 1:** Fluence of β- particles and equivalent dose rate at the sampling locations for the soil, plant and seed samples.

*Rosa spinosissima* L.			*Leymus angustus (*Trin*.) Pilg.*		
Sampling point no.	β, particles min^ − 1^ cm^ − 2^	γ, µSv h^ − 1^	Sampling point no.	β, particles min^ − 1^ cm^ − 2^	γ, µSv h^ − 1^
1	18	0.33	1	29	0.31
2	32	0.28	2	33	0.33
3	19	0.21	3	93	0.30
4	24	1.31	4	64	0.98
5	64	1.03	5	213	2.1
6	102	2.1	6	346	2.1
7	319	4.0	7	440	2.6
8	336	4.1	8	465	3.4
9	179	2.7	9	492	3.6
–	–	–	10	584	5.8

At the sampling points for wild-rye, the β-particle fluence values varied from 29–584 particles min^−1^ cm^−2^, and the EDR values ranged from 0.30–5.8 µSv h^−1^. Similarly, at the sampling points for *R. spinosissima,* the β-particle fluence values varied from 18–336 particles min^−1^ cm^−2^, and the EDR values ranged from 0.21–4.1 µSv h^−1^.

### Radionuclide contents in plants and soil

The activity concentrations of radionuclides in plants are presented in Tables 2 and 3.

**Table 2:** Activity concentrations of radionuclides in the samples of *Rosa spinosissima* L.

Sampling point no.	Activity concentrations of radionuclides, Bq kg^–1^					
	^90^Sr	^239 + 240^Pu	^137^Cs	^241^Am	^60^Co	^152^Eu
1	(1.2 ± 0.2)×10^4^	<0.02	31 ± 6	<0.2	<0.3	<2.2
2	(7.2 ± 1.1)×10^3^	0.21 ± 0.09	(2.0 ± 0.4)×10^3^	<0.5	<0.5	<3.0
3	(3.4 ± 0.5)×10^3^	0.24 ± 0.11	83 ± 17	<0.3	<0.4	<2.3
4	(1.1 ± 0.2)×10^3^	0.05 ± 0.04	(7.2 ± 1.4)×10^4^	<0.8	<0.3	<1.6
5	(7.7 ± 1.2)×10^3^	0.18 ± 0.11	(4.7 ± 0.9)×10^4^	<0.8	<0.3	<1.9
6	(9.0 ± 1.4)×10^3^	0.16 ± 0.04	(3.6 ± 0.7)×10^3^	<0.6	<0.5	<3.0
7	(1.4 ± 0.2)×10^4^	0.09 ± 0.04	(1.9 ± 0.4)×10^4^	<0.9	<0.8	<2.7
8	(1.5 ± 0.2)×10^4^	0.05 ± 0.04	(1.4 ± 0.3)×10^3^	<0.5	<2.0	<2.7
9	(1.8 ± 0.3)×10^4^	0.11 ± 0.05	(1.9 ± 0.4)×10^4^	<0.8	<0.4	<2.1

**Table 3:** Activity concentrations of radionuclides in the samples of *Leymus angustus* (Trin.) Pilg.

Sampling point no.	Activity concentrations of radionuclides, Bq kg^–1^					
	^90^Sr	^239 + 240^Pu	^137^Cs	^241^Am	^60^Co	^152^Eu
1	(5.2 ± 0.8)×10^3^	<0.09	(4.8 ± 1.0)×10^4^	<1.4	<0.7	<3.9
2	(6.1 ± 0.9)×10^3^	<0.06	(2.3 ± 0.5)×10^4^	<0.9	<0.5	<3.1
3	(3.4 ± 0.5)×10^4^	0.6 ± 0.2	(1.0 ± 0.2)×10^5^	<2.2	<1.0	<6.2
4	(1.4 ± 0.2)×10^4^	<0.04	(8.3 ± 1.7)×10^4^	<2.0	<0.8	<4.8
5	(4.7 ± 0.7)×10^4^	<0.1	(2.9 ± 0.6)×10^4^	<0.8	<0.5	<3.0
6	(1.5 ± 0.2)×10^4^	<0.05	(1.5 ± 0.3)×10^4^	<0.8	<0.7	<4.0
7	(2.0 ± 0.3)×10^4^	<0.06	(1.5 ± 0.3)×10^4^	<0.8	<0.8	<4.2
8	(1.3 ± 0.2)×10^3^	<0.05	(1.3 ± 0.3)×10^3^	<0.4	<0.5	<2.8
9	(2.4 ± 0.4)×10^4^	<0.03	(5.1 ± 1.0)×10^4^	<0.9	<0.5	<2.5
10	(1.7 ± 0.2)×10^4^	<0.08	160 ± 30	<0.4	<0.5	<2.7

At all sampling points, the activity concentrations of ^241^Am, ^60^Co, ^152^Eu, ^239+240^Pu in the plant samples either did not exceed or were close to their MDAs. However, a different trend was observed for ^137^Cs and ^90^Sr, whose activity concentrations in plants ranged within n × 10^3^ to n × 10^4^ Bq kg^-1^. Thus, the activity concentrations of ^137^Cs and ^90^Sr in plants were significantly higher than those of the other radionuclides. Therefore, these were the isotopes from which the plants received the highest doses.

The activity concentrations of radionuclides in the soil from the sampling locations for seeds and plants are listed in Tables 4 and 5.

**Table 4:** Activity concentrations of radionuclides in the soil at the sampling locations for *Rosa spinosissima* L.

Sampling point no.	Activity concentrations of radionuclides, Bq kg^–1^					
	^90^Sr	^239 + 240^Pu	^137^Cs	^241^Am	^60^Co	^152^Eu
1	99 ± 15	1.7 ± 0.6	(2.0 ± 0.4)×10^3^	<0.8	<0.6	<3.2
2	290 ± 40	1.3 ± 0.8	810 ± 160	<0.9	<0.6	<3.1
3	760 ± 110	8.2 ± 2.5	(2.4 ± 0.5)×10^3^	<0.8	<0.6	<3.6
4	(2.4 ± 0.4)×10^3^	<0.4	(2.5 ± 0.5)×10^5^	<3.0	<0.5	<2.9
5	(1.6 ± 0.2)×10^3^	<0.3	(6.4 ± 1.3)×10^3^	<1.4	<0.7	<3.2
6	(1.2 ± 0.2)×10^4^	2.3 ± 0.8	(1.2 ± 0.2)×10^4^	<2.1	<1.0	<6.8
7	(2.3 ± 0.3)×10^4^	29.1 ± 3.8	(2.3 ± 0.5)×10^5^	<7.6	<1.3	<7.1
8	(3.4 ± 0.5)×10^4^	3.9 ± 1.4	(1.1 ± 0.2)×10^5^	<6.9	<2.7	<15
9	(2.8 ± 0.4)×10^4^	13 ± 3	(1.8 ± 0.4)×10^5^	<7.3	<3.1	<9.5

**Table 5:** Activity concentrations of radionuclides in the soil at the sampling locations for *Leymus angustus* (Trin.) Pilg.

Sampling point no.	Activity concentrations of radionuclides, Bq kg^-1^					
	^90^Sr	^239 + 240^Pu	^137^Cs	^241^Am	^60^Co	^152^Eu
1	(2.8 ± 0.6)×10^3^	15.6 ± 2.5	(2.5 ± 0.5)×10^4^	<1.7	<0.4	22 ± 4
2	380 ± 170	1.9 ± 0.8	280 ± 60	<0.5	<0.4	<4.8
3	650 ± 190	0.9 ± 0.5	800 ± 160	<1.0	<0.7	<3.4
4	(3.7 ± 0.6)×10^3^	12.3 ± 1.7	(4.9 ± 1.0)×10^4^	<3.8	<1.6	16 ± 3
5	(2.4 ± 0.3)×10^4^	10.1 ± 1.5	(1.8 ± 0.4)×10^5^	<7.1	<1.5	<8.1
6	580 ± 170	2.9 ± 0.8	860 ± 170	<0.8	<1.1	<2.8
7	(5.3 ± 0.8)×10^3^	21 ± 3	(2.6 ± 0.5)×10^5^	<12	<1.6	<8.9
8	(1.8 ± 0.2)×10^4^	18.1 ± 3.6	(6.7 ± 1.3)×10^4^	<2.8	<0.7	<3.9
9	(1.9 ± 0.2)×10^4^	2.3 ± 0.8	(2.1 ± 0.4)×10^4^	<1.9	<0.7	<3.4
10	(2.4 ± 0.3)×10^4^	4.6 ± 1.2	(9.9 ± 2.0)×10^4^	<5.3	<1.2	<8.0

In the soil samples at the sampling points for *R. spinosissima* the activity concentration of ^90^Sr, ^239+240^Pu, ^137^Сs and ^152^Eu varied from 99 to 3.4 × 10^4^, 1.3 to 29.1, 810 to 2.5 × 10^5^ and <4.8 to 22 Bq kg^–1^, respectively. Similarly, in the soil samples at the sampling points for *L. angustus* the activity concentrations of ^90^Sr, ^239 + 240^Pu, ^137^Сs, and ^152^Eu varied from 380 to 2.4 × 10^4^, 1.9 to 21, 280 to 2.6 × 10^5^ and<4.8 to 22 Bq kg^–1^, respectively. No values of the activity concentrations of ^241^Am and ^60^Co were derived for the soil, where the plants of interest are growing. The values of the activity concentration of ^152^Eu are single and do not exceed 22 Bq kg^-1^.

### Assessment of the radiation burden on plants

The rates of radiation doses absorbed from internal and external exposures were calculated based on the activity concentrations of radionuclides in the soil and plant samples. The calculation results are listed in Table 6.

**Table 6:** Radiation doses absorbed by the plant species of interest.

*Rosa spinosissima* L.				*Leymus angustus* (Trin.) Pilg.			
Sampling point no	D_internal_, µGy/day	D_external_, µGy/day	D_total_, µGy/day	Sampling point no	D_internal_, µGy/day	D_external_, µGy/day	D_total_, µGy/day
1	192	5	197	1	226	67.6	293
2	131	2	133	2	151	0.786	152
3	55	53	108	3	748	2.19	750
4	579	550	1129	4	450	132	583
5	490	14	504	5	663	486	1150
6	172	27	199	6	231	2.35	233
7	373	506	879	7	291	702	993
8	251	242	493	8	20.1	181	201
9	437	396	833	9	462	56.7	518
–	–	–	–	10	205	267	472

It can be seen that the total radiation dose absorbed by *R. spinosissima* and *L. angustus* ranged from 108–1.129 µGy/day and 152–1150 µGy/day, respectively.

### Content of chemical elements in plants

The elemental contents of the two plant species are listed in Tables 7 and 8. At a few sampling points, there was a minor excess in the average concentrations of elements, such as cesium, cadmium, and uranium, in the ash of land plants [20]. Based on the results obtained, the concentrations of V, Cr, Mn, Co, Cu, Ni, Zn, As, Pb and Sr in all samples were lower than the corresponding mean concentrations of these elements in the ash of land plants, which made it possible to exclude the impact of these heavy elements on the cytogenetic variability of the plants of interest. In summary, the elemental content was not expected to have a toxic effect on the plants of interest.

**Table 7:** Contents of chemical elements in the samples of *Rosa spinosissima* L.

Elements	Sampling point no.									[20]*
	1	2	3	4	5	6	7	8	9	
	Contents of elements, mg kg^–1^ (ash)									
**V**	1.5 ± 0.2	1.0 ± 0.1	1.3 ± 0.2	1.8 ± 0.3	1.6 ± 0.2	1.5 ± 0.2	2.0 ± 0.3	1.7 ± 0.2	2.1 ± 0.3	30
**Cr**	4.8 ± 0.6	3.6 ± 0.5	4.7 ± 0.6	4.7 ± 0.7	5.5 ± 0.7	4.1 ± 0.5	6.3 ± 0.8	5.2 ± 0.7	6.5 ± 0.8	35
**Mn**	3,600 ± 470	750 ± 95	1,200 ± 150	360 ± 50	470 ± 60	860 ± 110	770 ± 95	910 ± 115	690 ± 90	4100
**Co**	0.6 ± 0.08	0.6 ± 0.07	0.7 ± 0.08	0.6 ± 0.08	0.7 ± 0.09	0.6 ± 0.07	0.7 ± 0.09	0.7 ± 0.09	0.9 ± 0.1	1
**Ni**	4.2 ± 0.6	5.7 ± 0.7	4.5 ± 0.6	4.6 ± 0.6	5.3 ± 0.7	3.8 ± 0.5	4.4 ± 0.6	5.1 ± 0.7	4.9 ± 0.6	40
**Cu**	28 ± 4	23 ± 3	29 ± 4	46 ± 6	40 ± 5	45 ± 6	37 ± 5	25 ± 3	33 ± 4	160
**Zn**	160 ± 20	200 ± 25	170 ± 20	290 ± 40	190 ± 25	170 ± 20	220 ± 30	140 ± 15	210 ± 25	600
**As**	0.9 ± 0.1	0.8 ± 0.1	0.8 ± 0.1	1.1 ± 0.2	0.8 ± 0.1	0.8 ± 0.1	0.9 ± 0.1	0.9 ± 0.1	1.0 ± 0.1	3
**Sr**	880 ± 120	790 ± 100	970 ± 120	550 ± 75	480 ± 60	660 ± 85	680 ± 90	730 ± 90	700 ± 90	700
**Cd**	0.99 ± 0.15	0.4 ± 0.05	1.1 ± 0.11	0.3 ± 0.1	0.3 ± 0.1	0.7 ± 0.1	1.2 ± 0.2	0.7 ± 0.08	0.8 ± 0.1	0.7
**Cs**	0.2 ± 0.03	1.2 ± 0.1	1.2 ± 0.1	17 ± 2	9.3 ± 1.0	0.85 ± 0.11	3.3 ± 0.4	0.45 ± 0.06	3.3 ± 0.4	3
**Pb**	2.1 ± 0.3	1.4 ± 0.2	1.9 ± 0.2	2.0 ± 0.3	1.5 ± 0.2	1.7 ± 0.2	1.8 ± 0.2	2.3 ± 0.3	2.7 ± 0.3	25
**U**	1.3 ± 0.2	0.5 ± 0.1	0.1 ± 0.02	0.7 ± 0.1	0.3 ± 0.1	0.8 ± 0.1	1.4 ± 0.2	1.6 ± 0.3	0.6 ± 0.1	0.4

Note: * –mean concentration of elements in the ash of terrestrial vegetation [10].

**Table 8:** Content of chemical elements in the samples of *Leymus angustus* (Trin.) Pilg.

Elements	Sampling point No.										[20]*
	1	2	3	4	5	6	7	8	9	10	
	Contents of elements, mg kg^–1^ (ash)										
**V**	0.9 ± 0.1	<0.03	0.9 ± 0.1	1.6 ± 0.2	0.8 ± 0.1	1.3 ± 0.2	1.3 ± 0.2	4.6 ± 0.6	1.2 ± 0.2	1.2 ± 0.2	30
**Cr**	2.3 ± 0.3	<0.02	2.6 ± 0.3	8.0 ± 1.0	2.8 ± 0.4	3.4 ± 0.5	3.5 ± 0.5	9.8 ± 1.2	3.6 ± 0.5	3.5 ± 0.5	35
**Mn**	240 ± 30	15 ± 2	160 ± 20	440 ± 50	80 ± 10	145 ± 20	130 ± 20	330 ± 40	50 ± 7	85 ± 11	4,100
**Co**	0.3 ± 0.03	<0.03	0.3 ± 0.04	0.5 ± 0.07	0.2 ± 0.03	0.3 ± 0.04	0.6 ± 0.08	0.9 ± 0.1	0.3 ± 0.04	0.3 ± 0.04	10
**Ni**	1.6 ± 0.2	<0.03	1.9 ± 0.3	4.5 ± 0.6	2.1 ± 0.3	1.8 ± 0.2	3.0 ± 0.4	3.8 ± 0.5	2.0 ± 0.3	2.1 ± 0.3	40
**Cu**	13 ± 2	<0.02	10 ± 1	31 ± 4	17 ± 2	15 ± 2	12 ± 1	24 ± 3	15 ± 2	15 ± 2	160
**Zn**	100 ± 10	5.1 ± 0.7	120 ± 15	380 ± 50	110 ± 10	53 ± 7	120 ± 10	140 ± 20	89 ± 11	55 ± 7	600
**As**	0.4 ± 0.1	<0.003	0.4 ± 0.1	0.7 ± 0.1	0.4 ± 0.1	0.6 ± 0.1	0.8 ± 0.1	1.5 ± 0.2	0.6 ± 0.1	0.5 ± 0.1	3
**Sr**	220 ± 30	29 ± 4	250 ± 30	490 ± 60	130 ± 10	190 ± 20	250 ± 30	300 ± 40	270 ± 30	230 ± 30	700
**Cd**	0.3 ± 0.04	<0.003	0.2 ± 0.03	0.9 ± 0.1	0.3 ± 0.03	0.2 ± 0.02	0.8 ± 0.1	0.9 ± 0.1	0.4 ± 0.06	0.2 ± 0.03	0.7
**Cs**	7.5 ± 0.95	<0.003	9.5 ± 1.2	26 ± 3	2.5 ± 0.3	1.3 ± 0.1	1.4 ± 0.1	1.5 ± 0.1	8.3 ± 1.0	0.4 ± 0.05	3
**Pb**	0.9 ± 0.1	<0.003	1.3 ± 0.2	1.3 ± 0.2	0.6 ± 0.1	1.0 ± 0.1	0.7 ± 0.1	4.3 ± 0.5	1.0 ± 0.1	1.1 ± 0.1	25
**U**	0.1 ± 0.02	<0.002	0.3 ± 0.04	0.4 ± 0.1	0.4 ± 0.05	0.7 ± 0.1	0.9 ± 0.1	1.6 ± 0.2	2.1 ± 0.3	0.7 ± 0.1	0.4

Note: * – mean concentration of elements in the ash of terrestrial vegetation [20].

### Cytogenetic indicators in plants

#### Range of cytogenetic aberrations.

**Fig 2:**
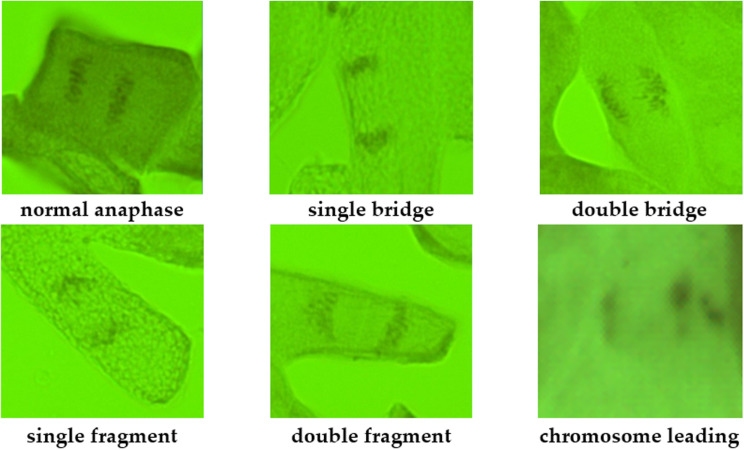
Cytogenetic abnormalities in the ana-telophase cells of *Rosa spinosissima* L.

During the cytogenetic analysis, various types of aberrations were discovered in the *R. spinosissima* population, including single and double bridges, single and double fragments, and mitotic disorders (chromosome lags and leads) (Fig 2). In addition, several types of aberrations were simultaneously observed in certain cells.

**Fig 3:**
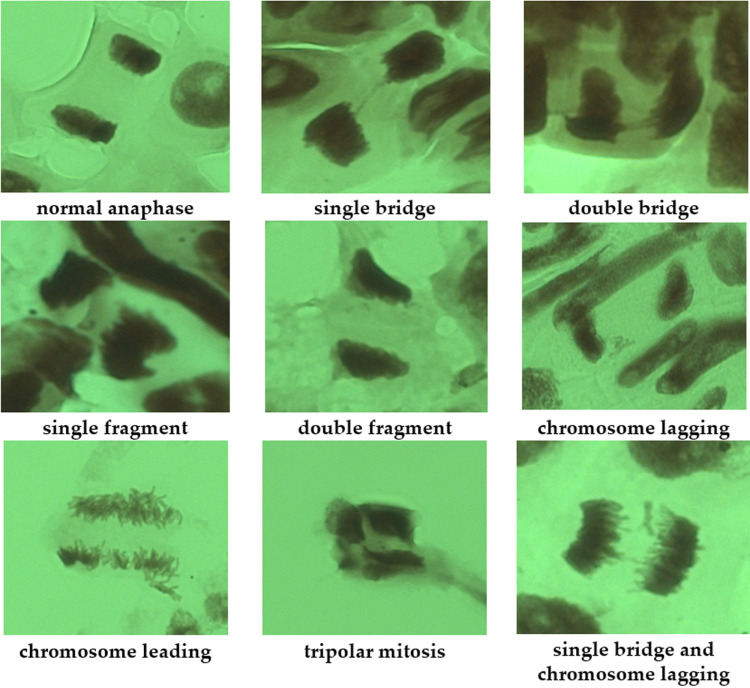
Cytogenetic abnormalities in the ana-telophase cells of *Leymus angustus* (Trin.) Pilg.

In the cytogenetic analysis of *L. angustus* the following aberrations were observed: single and double bridges, single and double fragments and mitotic aberrations (chromosome lags, leads and tripolar mitoses) (Fig 3).

In some cells, several types of aberrations were detected simultaneously. Chromosomal, chromatid and genomic mutations were also identified. *Leymus* similar to the plant species analysed in our earlier studies [5–7], belongs to the family of cereals but to a different genus. The main contribution to the formation of cytogenetic effects in all studied species of gramineous plants was made by double bridges and fragments, proving the radiative nature of the observed effects. To assess the possible impact of the radiological conditions at the ‘Degelen’ site, the frequency and range of cytogenetic disorders in the populations of the studied plant species were estimated. Data on the frequency of aberrations of various types and their relative contributions to the range of cytogenetic aberrations in *R. spinosissima* listed in Table 9.

**Table 9:** Frequency and range of cytogenetic aberrations in the germinates of *Rosa spinosissima* L.

Sampling point no.s	Viewed and examined			Number of aberrations							Ana-telophase cells with chromosomale aberrations, % ± SE
	Number of samples	Number of ana-telophase cells	Aberrant cells	m^Ι^	m^ΙΙ^	f^Ι^	f^ΙΙ^	g	s	Total number of aberrations	
1	12	252	4	1	–	1	1	1	–	4	1.6 ± 0.8
2	8	103	2	1	1	–	–	–	–	2	2.0 ± 1.4
3	10	161	3	1	1	1	–	–	–	3	1.9 ± 1.1
4	5	88	6	2	2	1	–	–	2	7	6.8 ± 2.7
5	32	507	12	3	3	1	2	2	1	12	2.4 ± 0.7
6	17	292	6	3	3	–	–	–	–	6	2.0 ± 0.8
7	8	109	5	1	2	–	–	2	–	5	4.6 ± 2.0
8	15	373	11	4	2	3	–	1	1	11	3.0 ± 0.9
9	6	106	6	3	2	1	–	–	–	6	5.7 ± 2.3

Note: f^Ι^ – single fragments; f^ΙΙ^– double fragments; m^Ι^ – single bridges; m^ΙΙ^ – double bridges; g– chromosome lags;

s – chromosome leads; “-” – no changes; and SE ‒ standard error.

Statistically significant difference from the minimum dose point: * *p* < 0.05.

Table 10 presents the cytogenetic indices of *L. angustus*.

**Table 10:** Frequency and range of cytogenetic aberrations in the germinantes of *Leymus angustus* (Trin.) Pilg.

Sampling point no.	Viewed and examined			Number of aberrations								Ana-telophase cells with chromosomal aberrations, % ± SE
	Number of samples	Number of ana-telophase cells	Aberrant cells	m^Ι^	m^ΙΙ^	f^Ι^	f^ΙΙ^	g	s	3^р^	Total number of aberrations	
1	48	790	13	3	6	1	–	1	2	–	13	1.6 ± 0.4
2	18	689	12	4	6	–	1	1	–	–	12	1.7 ± 0.5
3	31	953	33	13	13	2	2	3	1	–	34	*3.5 ± 0.6
4	49	1,217	63	7	36	8	1	6	3	2	63	*5.2 ± 0.6
5	70	1,644	70	15	42	4	1	4	3	1	70	*4.3 ± 0.5
6	60	1,702	26	8	9	3	2	2	1	1	26	1.5 ± 0.3
7	86	1,746	58	14	34	2	1	5	2	–	58	*3.3 ± 0.4
8	38	780	28	13	10	–	–	3	4	–	30	*3.6 ± 0.6
9	27	406	22	5	12	–	1	2	2	–	22	*5.4 ± 1.1
10	33	811	37	12	20	1	–	2	1	1	37	*4.6 ± 0.7

Note: f^Ι^ – single fragments; f^ΙΙ^ ‒ double fragments; m^Ι^ – single bridges; m^ΙΙ^ – double bridges; g ‒ chromosome lags; s – chromosome leads; “-” ‒ no changes; and SE ‒ standard error.

**Fig 4:**
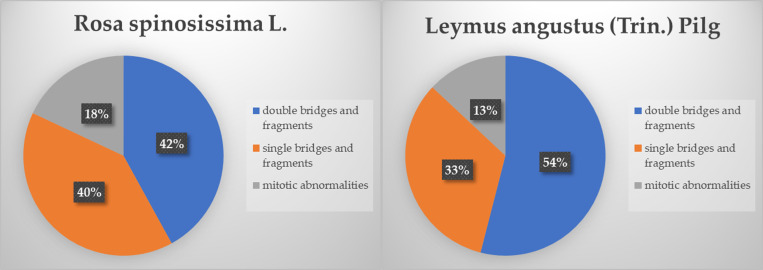
Range of cytogenetic aberrations.

Statistically significant difference from the minimum dose point: * *p* < 0.05.The analysis of mutations in the *R. spinosissima* population revealed at predominance of structural changes in the chromosomes. The greatest contribution was made by chromosomal aberrations (double bridges and fragments constituted 42% of the total number of mutations), chromatid aberrations (single bridges and fragments constituted 40%). Mitotic abnormalities constituted 18%, which is much lower than the proportions of the other types of abnormalities (Fig 4).

**Fig 5:**
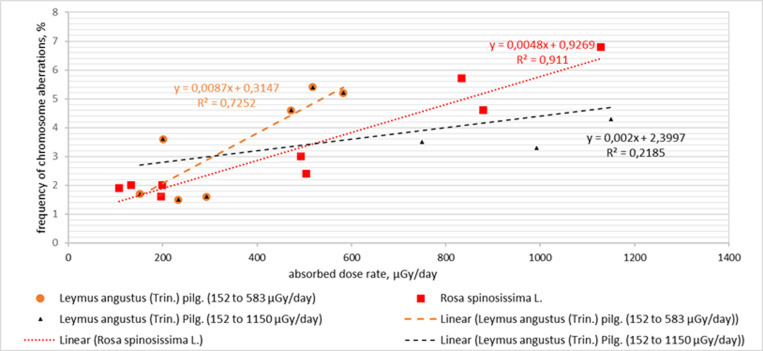
Relationship between the frequency of aberrant cells in plants and the absorbed radiation dose.

The bulk of the range of structural mutations in the population of *L. angustus* was constituted by chromosomal aberrations (double bridges and fragments) induced by the radiation, which were more than half of the cytogenetic effects – at 54%, chromatid disorders (single bridges and fragments) were approximately 33% and mitotic disorder (chromosome leads, lags and tripolar mitosis) made the least contribution of only 13% (Fig 3). In *L. angustus* populations growing in the most contaminated areas, the peak frequency of aberrant cells was observed to reach 5.4 ± 1.1%. In relatively less contaminated areas, the frequency of aberrant cells ranged within 1.5 ± 0.3%, which is close to the typical frequency of aberrant cells in many wild and cultivated cereals (0.5–1.0%) [4]. Previous research into the cytogenetic effects of chronic radiation exposure on *Stipa capillata L.* growing at the test site ‘4A’ reported that the peak frequency of aberrant cells reaches 5.0% [7], and in the populations of *Koeleria gracilis* Pers. from the same site, it reaches 15% [5,6]. Double bridges and fragments are the main contributors to the generation of cytogenetic effects in all species of gramineous plants, proving the radiative nature of the observed effects. Based on these findings, the relationship between the frequency of aberrant cells and the dose absorbed by plants was estimated. Based on these findings, the relationship between the frequency of aberrant cells and the dose absorbed by plants was estimated. The frequencies of aberrant cells in the plants of interest depending on the absorbed radiation dose, and are depicted in Fig 5.

As the exposure to the radiation dose increased in the interval from the natural background to 1,129 µGy/day, the frequency of aberrant cells was found to increase in direct proportion in the *R. spinosissima* population. This effect became linear and was because of a lasting impact of β- and γ-rays in the study area. In case of *L. angustus*, the frequency of cytogenetic damage demonstrated significant variability, both in a fairly narrow range of low radiation dose rates and with higher values, which may indicate no strong correlation between the frequency of aberrant cells and the radiation dose rate. The tendency towards such relationship is attributable to dose interval 152–583 µGy/day, at which a direct relationship was traceable between the frequency of aberrant cells and the absorbed radiation dose ranging from 0–583 µGy/day. These findings enable the analysis of cytogenetic effects in the *R. spinosissima* population versus *L. angustus* population for better insight into the effects of various stressors on plants. The plant species studied belong to different families and genera, as *L. angustus* is a herbaceous plant species, whereas *R. spinosissima* is a brushwood species. The seeds of *L. angustus* are twice as big as those of *R. spinosissima*, and its ana-telophase chromosomes in the mitosis phase have more chromosomes than in *R. spinosissima*. The frequency of cytogenetic aberrations in *R. spinosissima* at the maximum dose point was higher (7.0%) than in *L. angustus* (5.4%). In contrast, *L. angustus* exhibited tripolar mitoses, whereas *R. spinosissima* exhibited no such mitotic abnormalities, even though both plant species were collected from the same ecosystem, contaminated with a water stream from adit 176. *Leymus angustus* exhibited a significant number of chromosomal aberrations, indicating an elevated radiosensitivity of this species relative to that of *R. spinosissima*. Thus, a linear relationship was established between the increase in the frequency of aberrant cells and the increase in the rate of absorbed radiation dose in *R. spinosissima* populations. This effect was specific to the entire dose range. However, no linear relationship was observed for *Leymus angustus* (trin.) Pilg. population between the increase in the frequency of aberrant cells and increase in the absorbed dose rate for the entire absorbed dose range. A linear relationship of the growth in the frequency of aberrant cells to the increase in the absorbed dose rate is only noted to be up to 583 µGy/day (Fig 5) Perhaps, the dose range from 583 µGy/day to 1,129 µGy/day is not sufficient for a stable growth of the frequency of chromosome aberrations. In summary, radiosensitivity of this species remains ambiguous. On the one hand, *R. spinosissima* exhibited a permanent linear growth in the frequency of aberrant cells as the rate of the absorbed radiation dose increased in the entire range of the absorbed radiation dose in question, and on the other, *L. angustus* responded relatively more acutely up to 583 µGy/day, and later on, the frequency of aberrant cells ceased to increase with the increase in the rate of the absorbed radiation dose. This may be associated with the diverse mechanisms of plant responses to stressors, and this must be considered when choosing bioindicators for various biomonitoring purposes. Earlier research into the impact by radionuclide contamination on the morpho-anatomical and anatomical indicators of the plant *Calamagróstis epigéjos* growing at the Degelen site did not reveal any influence at the cellular level in the dose range from 40–760 µGy/day [21]. In our study, at the cytogenetic level, a relationship was established between the frequency of chromosomal aberrations and the quantity of absorbed dose in *R. spinosissima* (Fig 5).

## Conclusions

Based on the results obtained in this study the radiation dose absorbed by the two plant species ranged from 108–1,150 µGy/day, depending on the sampling points for the plant samples. The main exposure dose received by plants was from ^90^Sr and ^137^Cs. In both plant species, chromosomal aberrations (double bridges and double fragments) were the main contributors to the range of cytogenetic effects. For *Rosa spinosissima L.*, chromosomal aberrations accounted for 42%, whereas, for *Leymus angustus* (Trin.) Pilg., they accounted for 54%. A linear relationship was established between the increase in the frequency of aberrant cells and the increase in the rate of radiation dose absorption in *R. spinosissima* for the entire range of the absorbed doses in question up to 1,129 µGy/day and in *L. angustus* for the range of absorbed doses from 152–583 µGy/day.
